# Impact of the Fogarty Training Program on Trainee and Institutional Research Capacity Building at a Government Medical College in India

**DOI:** 10.5334/aogh.2932

**Published:** 2020-07-28

**Authors:** Gauri Dhumal, Andrea DeLuca, Ajay Chandanwale, Dileep Kadam, Samir Joshi, Aarti Kinikar, Nikhil Gupte, Vidya Mave, Amita Gupta, Nishi Suryavanshi, Robert C. Bollinger

**Affiliations:** 1 Byramjee Jeejeebhoy Government Medical College/Johns Hopkins Clinical Trials Unit, Pune, Maharashtra, IN; 2 Department of Health Policy and Management, Johns Hopkins Bloomberg School of Public Health, MD, US; 3 Department of Orthopedics, Byramjee Jeejeebhoy Government Medical College/Sassoon General Hospital, Pune, Maharashtra, IN; 4 Department of Medicine, Smt. Kashibai Navale Medical College and General Hospital, Pune, Maharashtra, IN; 5 Department of ENT, Byramjee Jeejeebhoy Government Medical College/Sassoon General Hospital, Pune, Maharashtra, IN; 5 Department of Pediatric, Byramjee Jeejeebhoy Government Medical College/Sassoon General Hospital, Pune, Maharashtra, IN; 7 Medicine and Public Health, Division of Infectious Diseases, Johns Hopkins University School of Medicine, MD, US; 8 Medicine, Public Health, and Nursing, Division of Infectious Diseases, Johns Hopkins University School of Medicine, MD, US

## Abstract

**Background:**

Strengthening health research is essential to inform public health policies. However, few research training programs have systematically measured their impact on capacity building and most evaluations have been limited to reporting of individual trainee metrics. Hence, we conducted an evaluation of the impact of a five-year training program focused on building both trainee and institutional research capacity at a public medical college in India.

**Methods:**

Quantitative and qualitative methods were used to assess the individual and institutional research capacity building of a five-year HIV-TB research training program at Byramjee Jeejeebhoy Government Medical College in Pune, India, supported by the US National Institutes of Health, Fogarty International Center. In addition to documentation of the number of trainee research projects initiated, the number of research papers produced by the Fogarty Scholars (FSs) available on PubMed was calculated. The institutional impact of this program was assessed by documentation of research training activities conducted by the FSs, as well as by surveys and in-depth interviews conducted at the beginning and end of the program.

**Results:**

Twenty-one mid-level BJGMC faculty were provided training in HIV-TB research competencies. Between 1 April 2014 and 1 April 2019, 13 of these FSs designed and implemented new IRB-approved research studies and contributed to 49 PubMed listed research papers, including 11 first-authored manuscripts. FSs also conducted 36 journal club discussions, mentored 58 student research projects and conducted 5 institutional research method workshops. Pre- and-post-program surveys and in-depth interviews documented a perceived increase in institutional research capacity, particularly in TB research (epidemiology, clinical research, laboratory research). The impact of the Fogarty Training Program on institutional scientific output was perceived to be marginally improved.

**Conclusion:**

The Fogarty Training Program had a significant impact on building individual research capacity. To sustain this impact beyond the five years of Fogarty support, additional governmental and institutional resources, the establishment of dedicated space for faculty research and protected faculty time for research are needed. These findings can inform the design and implementation of future health research capacity building initiatives.

## Background

India has the highest TB burden in the world, accounting for 27% of all new cases [1]. India also ranks third globally in absolute HIV burden, with 2.1 million people living with HIV (PLHIV) and 86,000 new HIV infections in 2017 [2]. PLHIV are 20–30 times more likely to develop TB, and TB is the leading cause of death among PLHIV [3]. Capacity development for optimizing the management of HIV and TB in India can expand the ability to study important clinical research questions, such as epidemiological questions focused on risk of development of drug-susceptible and drug-resistant TB [1,4,5]. Health care professionals are the ones who are directly involved in the HIV-TB management; hence, reinforcing their research capacity in this arena is very essential [6].

Augmenting the capacity to carry out health research is urgently needed for improving global health services and the health of the population [7,8]. In low- and middle-income countries (LMIC), development of local scientific leadership and research capacity is a crucial factor to improve the research capacity at local level [9,10].

Strengthening human resources for health research (HRHR) is crucial to inform public health policies [11,12]. A number of research training programs by the World Health Organization (WHO), the National Institute of Health (NIH) and Fogarty International Centre (FIC) have highlighted the importance of training for health researchers. Other programs like Enhancing Research Capacity, Danish Ministry of Foreign affairs (ENRECA) and the National Institute for Health, Fogarty International Centre (US NIH FIC), USA, have addressed the need for training programs to enhance research capacity [12,13,14]. However, few programs have systematically measured the impact of their training on capacity building, and most evaluations have been limited to reporting of individual trainee metrics. Measuring institutional research capacity building is challenging [14]. We conducted an evaluation of the impact of a five-year training program focused on building both trainee and institutional research capacity at a public medical college in India.

## Methods

### Design

Mixed-methods analyses were conducted, which included quantitative surveys and qualitative in-depth interviews at the beginning and at the end of the program.

### The Fogarty HIV-TB Research Training Program

The Fogarty HIV-TB Research Training Program was initiated in April 2014. Strengthening and assessment of institutional research capacity was set as the foremost goal by the NIH program supporting this project. The training program was designed by leadership of JHU and BJGMC. The leadership of BJGMC defined HIV/TB research capacity as the priority for the program. The program also leveraged successful methods and lessons learned from previous Fogarty HIV training programs. Eligible candidates submitted an application that outlined their proposed research project, as per their interest area and expertise. The program established a Training Advisory Committee (TAC), that included experienced HIV/TB experts from India and the US, including Indian experts not affiliated with BJGMC. All the proposals were reviewed and ranked by the TAC and accordingly the candidates were selected for the training program.

A total of 21 faculty scholars were recruited from Byramjee Jeejeebhoy Government Medical College (BJGMC), Pune, India, and trained over the subsequent five years, in three groups. The program was designed to recruit three sequential groups of scholars over the five-year training grant period. The annual program budget could only support a limited number of trainees to attend the Hopkins Summer Institute. Therefore, three separate training cohorts were recruited. Group 1 included five scholars who initiated their training in 2014, Group 2 included six scholars who initiated training in 2015 and Group 3 included 10 scholars who initiated training in 2017. The three groups of Fogarty Scholars had varied training exposure time period to the Fogarty program, once they returned to India: Group 1 was exposed for five years, Group 2 for four years and Group 3 for one year. Of the 21 scholars, 11 were female, median age was 42 years (IQR 32–51), 15 had completed an MD degree in their respective specialities (Medicine, Pediatrics, Preventive and Social Medicines, Dermatology, Gynaecology, Microbiology, Pulmonary Medicine, Pharmacology, Anaesthesia, Radiology) with one scholar holding an additional PhD degree, five had an MS degree (Orthopedics, ENT, Surgery, Opthalmology) while one scholar held a PhD degree in Medical Biochemistry.

All Fogarty Scholars (FSs) began with an intensive three-week summer training course in epidemiology and biostatistics at the Johns Hopkins Bloomberg School of Public Health, Baltimore, which was delivered by JHU faculty. JHU faculty also provided on-site and remote mentoring of the scholars for their research projects, as well as participation in monthly journal clubs and research presentations. Distance-learning modules were supplemented with regular professional development activities at BJGMC, which included peer-supported educational programs (e.g., journal clubs).

The research competencies taught remotely covered a range of topics including human subject’s research compliance, Good Clinical Practice (GCP)/Human Subjects Research (HSR), and Introduction to Scientific Presentations, Paper Writing, Grant Writing, and Data Management. Fogarty scholars were asked to participate in and subsequently lead regular monthly group sessions with Hopkins and BJGMC faculty focused on helping them each develop a specific HIV-TB research study protocol. Once the scholars returned to their institution after the training program in the US, they spent on average, 1–2 hours per day working on their Fogarty study proposal development, data collection, and manuscript writing. At their institution, they were expected to obtain regulatory approval and to implement their research projects, as well as to analyze and publish their results in peer-reviewed journals deposited on PubMed Central. They were also expected to support additional institutional research capacity strengthening at BJGMC. This included engagement of additional BJGMC faculty and students in their research, development of monthly HIV-TB journal clubs and providing institutional research training. Fogarty Scholars usually spent 2–3 hours per month on institutional research capacity strengthening. To facilitate their efforts, Fogarty Scholars received regular guidance and input from Hopkins and BJGMC faculty mentors for these activities throughout the duration of the program.

### Population

#### Quantitative surveys

Prior to the award of the Fogarty grant, a research workshop was conducted in 2013 at B.J. Government Medical College, Pune, India, that included 61 global HIV-TB research experts from India and USA. At the beginning of the program, in April 2014, a quantitative survey was distributed to these 61 HIV-TB research experts, 54 of whom were considered independent of the Fogarty Program (i.e. not employed by or collaborating with BJGMC). At follow-up in February 2018, the same 61 experts plus the additional (20) Fogarty Scholars (total 81) were invited to participate in the final evaluation survey.

#### Qualitative In-depth Interviews

In April 2014, five of the 54 independent HIV-TB research experts who participated in the quantitative survey were also invited to participate in qualitative in-depth interviews at the beginning of the Fogarty program. Four were Indian and one was US based. The same five independent HIV-TB research experts were invited for follow-up interviews during December 2017–March 2018 (at the end of the program), of which four (three Indian and one US-based expert) agreed to be interviewed. In addition, all 21 Fogarty Scholars and 4 institutional leaders at BJGMC were also invited for in-depth interviews; 3 senior institutional leaders at BJGMC and 19 Fogarty Scholars agreed to be interviewed.

### Data

#### Individual trainee research publications

The names of all 21 Fogarty Scholars were searched using the PubMed author field to document the number of manuscripts listing them as a first or contributing author. The baseline scientific output of the BJGMC faculty who participated in the training program was defined as the number of publications attributed to them during the five years prior to the Fogarty Program, through a search from 31 March 2009 to 31 March 2014. This was compared to the number of publications attributed to Fogarty Scholars since initiation of the Fogarty Training Program, published between 1 April 2014 and 1 April 2019.

#### Individual trainee research proposals

The BJGMC Fogarty Program Manager (GD) maintained records of all research protocols developed by the Fogarty Scholars, as well as their IRB submissions and research grant submissions.

#### Trainee activities contributing to institutional research capacity

The Fogarty Scholars monthly journal club participation, their development and presentation of research training workshops for their peer faculty and students, their research collaborations that engaged other BJGMC faculty who were not part of the formal Fogarty training program, and their mentorship of BJGMC student research projects were also assessed.

#### A quantitative survey assessing institutional research capacity

The baseline and follow-up semi-structured electronic survey form included 15 questions that asked respondents to use a Likert Scale for the respondent’s self-assessment of the strength of their responses to rate BJGMC’s institutional research capacity in a number of specific areas, including overall faculty knowledge about basic research, TB clinical care, and TB epidemiology. (Annexure I; Survey questionnaire). Respondents were also asked to rate BJGMC’s TB lab capacity, scientific output, and linkages with government, as well as linkages with Indian and International TB experts.

#### Qualitative in-depth interviews assessing institutional research capacity

Following informed consent, one-on-one interviews were conducted by trained social scientists face-to-face, via phone, or via internet communication. The study social scientists used an interview guide (Annexure II A, B, C: In-depth interview guides), which focused on documenting the opinion and recommendations of the interviewees about the capacity of BJGMC to conduct HIV-TB research and the institutional training needs.

### Statistics

#### Quantitative analyses

Survey results were analyzed using descriptive statistics. A z-test was conducted to calculate the p-value for the comparison of the number of manuscripts published by Fogarty Scholars before and after Fogarty training program.

#### Qualitative analyses

Audio recordings of the in-depth interviews were transcribed verbatim. An experienced social scientist (GD) and the program director (AD) agreed on a final code set for analysis. Five major themes were addressed in the baseline and follow-up interviews including: 1) capacity of BJGMC to conduct HIV-TB research; 2) institutional needs for research capacity strengthening; 3) status of collaboration between BJGMC and other research institutions; 4) BJGMC’s ability to disseminate research results; and 5) barriers to conducting HIV-TB research at BJGMC. The follow-up interviews addressed two additional themes: 6) Fogarty Scholars’ view regarding the Fogarty training program and 7) recommendations to overcome institutional barriers to conducting HIV-TB research. Data were analyzed using structured thematic analysis by MaXQDA software version 12.

### Ethics

The study protocol was approved by the BJGMC ethics committee, India, and the Johns Hopkins University Institutional Review Board (JHU IRB), USA.

## Results

### Individual Trainee Research Capacity

During the five years prior to the initiation of the Fogarty program, 21 BJGMC faculty members contributed to 18 PubMed listed research publications, of which 8 manuscripts were first-authored by Fogarty Scholars. After the program, the same Fogarty Scholars contributed to 49 PubMed listed research publications from 1 April 2014 till 1 April 2019. Of these, 11 manuscripts were first-authored by Fogarty Scholars. There was a significant increase in the number of publications attributed to the Fogarty Scholars before and after the Fogarty training program (p < 0.0001). As of 1 April 2019, 13 of the 21 Fogarty Scholars had developed and initiated their own IRB-approved research protocol, of which six studies have been completed and the remaining are ongoing. One additional FS has obtained IRB-approval and study initiation is pending. Group 1 Fogarty Scholars received an NIH research grant supplement award for the research proposal developed collaboratively through the Fogarty program training. Currently, of the 21 Fogarty-trained faculty, 17 are still present at the institution. Three Fogarty scholars were subsequently transferred to other Indian government institutions, and one has left the institution. Eight scholars attended and presented abstracts at international conferences specifically related to HIV-TB. Along with these eight scholars, an additional two scholars presented their abstracts at national conferences.

### Quantitative Assessment of Institutional Research Capacity

Thirty-six monthly journal club activities were conducted at BJGMC by the Fogarty Scholars during the five-year training period. The Fogarty Scholars also prepared and conducted four annual research methodology workshops for other BJGMC faculty and students (Years 2–5). An additional research methodology workshop was conducted by the Fogarty Scholars for a national conference for undergraduate (UG) students held at BJGMC. Another indication of institutional research capacity strengthening was the development and initiation of 58 BJGMC student research projects that were supervised by the Fogarty Scholars. To date, five of these FS-mentored student projects have received an Indian Council of Medical Research (ICMR) student project funding award. In addition, the Fogarty Scholars have engaged 14 of their BJGMC faculty colleagues as publication co-authors, from nine different departments.

Survey responses were received from 22 (36%) of the 61 experts sent links at baseline and from 30 (37%) of 81 at follow up. As shown in Table 1, in 2014, HIV-TB research capacity at BJGMC was ranked as strong or very strong only in the area of TB clinical knowledge by most (>68%) of the respondents. In 2018, HIV-TB research capacity at BJGMC was ranked as strong or very strong by most of the respondents (≥60%) in all of the six listed categories, except for overall scientific output (only 27% of respondents ranked BJGMC as “strong” or “very strong”). In addition, most (>50%) of the baseline survey respondents identified BJGMC’s strong or strongest need for training in the areas of basic research knowledge (83.3%), TB lab skills (68.4%) and scientific output (75%) (e.g. manuscript and grant preparation). At the end of the program, most respondents (56.6%) continued to identify scientific output as a strong or the strongest need for additional training. Training in basic research and TB lab skills were no longer seen as significant needs by most survey respondents in 2018.

**Table 1:** Baseline and Follow-up Survey of HIV-TB Research Capacity at BJGMC.^1^

Respondents ranking strength of HIV-TB researchers at BJGMC n (%)												

Specific Research Capacity	Responses Received		Very Strong		Strong		Mediocre		Weak		Very Weak	

	2014	2018	2014	2018	2014	2018	2014	2018	2014	2018	2014	2018

Basic Research Knowledge	20	30	0 (0.0)	2 (6.7)	6 (30.0)	16 (53.3)	6 (30.0)	9 (30.0)	5 (25.0)	3 (10.0)	3 (15.0)	0 (0.0)
TB Lab Skills	21	30	1 (4.8)	2 (6.7)	9 (42.9)	21 (70.0)	10 (47.6)	7 (23.3)	0 (0.0)	0 (0.0)	1 (4.8)	0 (0.0)
TB Clinical Knowledge	22	29	2 (9.1)	4 (13.8)	13 (59.1)	23 (79.3)	7 (31.8)	2 (6.9)	0 (0.0)	0 (0.0)	0 (0.0)	0 (0.0)
TB Epidemiology Knowledge	21	30	0 (0.0)	3 (10)	8 (38.1)	20 (66.7)	9 (42.9)	7 (23.3)	4 (19.0)	0 (0.0)	0 (0.0)	0 (0.0)
Scientific Output	22	30	0 (0.0)	2 (6.7)	0 (0.0)	6 (20.0)	6 (27.3)	17 (56.6)	13 (59.1)	5 (16.7)	3 (13.6)	0 (0.0)
Linkage with Government	17	30	0 (0.0)	2 (6.7)	4 (23.5)	17 (56.7)	12 (70.6)	10 (33.3)	1 (5.9)	1 (3.3)	0 (0.0)	0 (0.0)
**Respondents Ranking for the areas requiring the most training n (%)**												

**Specific Research Capacity**	**Responses Received**		**Strongest need**		**Strong need**		**Moderate Need**		**Weak Need**		**Weakest Need**	

	**2014**	**2018**	**2014**	**2018**	**2014**	**2018**	**2014**	**2018**	**2014**	**2018**	**2014**	**2018**

Basic Research Knowledge	18	30	7 (38.9)	1 (3.3)	8 (44.4)	13 (43.3)	1 (5.6)	10 (33.3)	2 (11.1)	5 (16.7)	0 (0.0)	1 (3.3)
TB Lab Skills	19	28	3 (15.8)	0 (0.0)	10 (52.6)	9 (32.1)	4 (21.1)	14 (50.0)	2 (10.5)	2 (7.1)	0 (0.0)	3 (10.7)
TB Clinical Knowledge	19	30	2 (10.5)	1 (3.3)	3 (15.8)	6 (20.0)	9 (47.4)	9 (30.0)	4 (21.0)	12 (40)	1 (5.3)	2 (6.7)
TB Epidemiology Knowledge	20	29	4 (20.0)	2 (6.9)	4 (20.0)	8 (27.6)	9 (45.0)	8 (27.6)	3 (15.0)	7 (24.1)	0 (0.0)	4 (13.8)
Scientific Output	20	30	11 (55.0)	7 (23.3)	4 (20.0)	10 (33.3)	4 (20.0)	9 (30.0)	1 (5.0)	1 (3.3)	0 (0.0)	3 (10.0)
Linkage with Government	16	29	0 (0.0)	1 (3.4)	4 (25.0)	6 (20.7)	11 (68.8)	12 (41.4)	1 (6.2)	8 (27.6)	0 (0.0)	2 (6.9)

^1^ 2014: 61 TB experts sent survey, 22 responses received; 2018: 61 TB experts + 20 Fogarty Scholars sent survey, 30 responses received.

Another question in the survey assessed BJGMC’s capacity in specific research competencies (Table 2). As opposed to the baseline survey when BJGMC was not perceived as either “well” or “very well” positioned in any of these seven competency areas, at the end of the program, >70% of respondents ranked BJGMC as “well” or “very well” positioned in four of these seven areas: TB laboratory facilities, Good Clinical Laboratory Practices (GCLP), TB clinical research and the ethical conduct of research. Table 3 shows the survey responses to questions about BJGMC’s research linkages to five different stakeholder groups: other BJGMC faculty researchers, BJGMC medical students, Indian government organizations, other Indian TB researchers, and foreign TB researchers. At baseline, most respondents (range 40–70%) reported that the linkages of BJGMC’s TB researchers with all of these stakeholders were “poor”, “terrible”, or “not enough information to answer”. In contrast, the perceived linkages with all of these stakeholders had increased by the end of the training program.

**Table 2:** Baseline and Follow-up Survey of HIV-TB Research Capacity at BJGMC.^1^

Respondents Ranking Position of BJGMC to do TB Research with Regards to the following Parameters, n (%)														

Specific Research Capacity	Responses Received		Very Well Positioned, no Gaps		Well Positioned with Some Institutional Gaps		Somewhat Well Positioned with Institutional Gaps		Not Very Well Positioned with Significant Institutional Gaps		Badly Positioned with Major Institutional Gaps		Not Enough Experience to Say	

	2014	2018	2014	2018	2014	2018	2014	2018	2014	2018	2014	2018	2014	2018

TB Laboratory Facilities	20	28	0 (0.0)	4 (14.3)	7 (35.0)	18 (64.3)	4 (20.0)	4 (14.3)	1 (5.0)	2 (7.1)	1 (5.0)	0 (0.0)	7 (35.0)	0 (0.0)
Good Clinical Laboratory Practice	20	28	1 (5.0)	4 (14.3)	6 (30.0)	16 (57.1)	4 (20.0)	5 (17.9)	3 (15.0)	3 (10.7)	0 (0.0)	0 (0.0)	6 (30.0)	0 (0.0)
TB Clinical Research System	20	28	0 (0.0)	5 (17.9)	4 (20.0)	15 (53.6)	9 (45.0)	7 (25.0)	1 (5.0)	1 (3.5)	1 (5.0)	0 (0.0)	5 (25.0)	0 (0.0)
TB Epidemiologic Investigations	20	28	2 (5.0)	3 (10.7)	1 (5.0)	11 (39.3)	5 (25.0)	10 (35.7)	8 (40.0)	2 (7.1)	0 (0.0)	2 (7.1)	5 (25.0)	0 (0.0)
Ethical Conduct of Research	20	28	2 (10.0)	8 (28.6)	5 (25.0)	15 (53.6)	6 (30.0)	4 (14.3)	0 (0.0)	0 (0.0)	1 (5.0)	0 (0.0)	6 (30.0)	1 (3.6)
Data Management Systems	19	28	0 (0.0)	4 (14.3)	5 (26.3)	7 (25.0)	0 (0.0)	8 (28.6)	7 (36.8)	4 (14.3)	1 (5.3)	3 (10.7)	6 (31.6)	2 (7.1)
Research Dissemination Systems	20	28	0 (0.0)	3 (10.7)	1 (5.0)	4 (14.3)	4 (20.0)	12 (42.9)	7 (35.0)	6 (21.4)	2 (10.0)	2 (7.1)	6 (30.0)	1 (3.6)

^1^ 2014: 61 TB experts sent survey, 20 responses received; 2018: 61 TB experts + 20 Fogarty Scholars sent survey, 30 responses received.

**Table 3:** Baseline and Follow-up Survey of BJGMC’s TB Research Linkages to.^1^

Respondents Ranking Position of BJGMC to do TB Research with Regards to the following Parameters, n (%)														

	Responses Received		Excellent		Good		Fair		Poor		Terrible		Not Enough Information to Answer	

	2014	2018	2014	2018	2014	2018	2014	2018	2014	2018	2014	2018	2014	2018

Other BJGMC Researchers	20	28	0 (0.0)	5 (17.9)	4 (20.0)	11 (39.3)	2 (10.0)	11 (39.3)	1 (5.0)	0 (0.0)	1 (5.0)	0 (0.0)	12 (60.0)	1 (3.6)
BJGMC Medical Students	20	28	0 (0.0)	3 (10.7)	3 (15.0)	9 (32.1)	3 (15.0)	12 (42.9)	4 (20.0)	2 (7.1)	1 (5.0)	0 (0.0)	9 (45.0)	2 (7.1)
Government Bodies, including the RNTCP	20	29	0 (0.0)	2 (6.9)	4 (20.0)	17 (58.6)	3 (15.0)	4 (13.8)	4 (20.0)	3 (10.3)	2 (10.0)	0 (0.0)	7 (35.0)	3 (10.3)
Other Indian TB Researchers	19	29	0 (0.0)	1 (3.4)	4 (21.0)	12 (41.4)	3 (15.8)	8 (27.6)	4 (21.0)	5 (17.2)	2 (10.5)	0 (0.0)	6 (31.6)	3 (10.3)
Other Foreign TB Researchers	20	29	2 (10.0)	3 (10.3)	6 (30.0)	12 (41.4)	4 (20.0)	5 (17.2)	1 (5.0)	6 (20.7)	2 (10.0)	0 (0.0)	5 (25.0)	3 (10.3)

^1^ 2014: 61 TB experts sent survey, 20 responses received; 2018: 61 TB experts + 20 Fogarty Scholars sent survey, 29 responses received.

### Qualitative Interviews about Institutional and Trainee Research Capacity

Qualitative results were presented in themes. Each theme reflects a common issue expressed by multiple respondents. The most representative quote of each theme has been included in the results.

#### Theme 1: Capacity of BJGMC to conduct HIV-TB research

The baseline interviews of independent TB research experts (3) confirmed the baseline survey findings that capacity for HIV-TB research at BJGMC was limited, at the beginning of the Fogarty Program. One TB expert said,

I think there are a few people there who are capable of doing research, but my feeling is that they haven’t had much opportunity so far or much training or exposure.

Fourteen (53%) of the 26 interviewed at the end of the program confirmed the quantitative survey findings that HIV-TB research capacity had improved at BJGMC and that this increased capacity strengthening was a direct result of the Fogarty program. One TB expert said,

I have heard involvement at several meetings in both nationally and internationally and I have no doubt about that.

#### Theme 2: Need for research capacity strengthening

The baseline interviews confirmed the baseline survey findings that BJGMC faculty needed research training. One TB expert said,

There is no training in the medical curriculum in India on grant-writing or research methodology or anything like that, so people really have to pick up the skills on their own.

At follow-up, 8 (30%) of the 26 interviewed confirmed the quantitative survey findings that there continues to be an additional need for research capacity strengthening at BJGMC, particularly related to scientific output. One Fogarty Scholar said,

I still feel that I have to improve my writing skills.

#### Theme 3: Status of collaboration between BJGMC and other research institutions

At baseline, three of five interviewed confirmed the baseline survey findings that BJGMC research collaborations were primarily limited to a long-standing partnership with Johns Hopkins University. One of the TB experts said,

They have gained a lot of experience through their collaboration with Johns Hopkins group.

At follow-up, 22 (84%) of the 26 people interviewed confirmed the quantitative survey findings that research collaborations have improved between BJGMC and other institutions in India. One of the BJGMC leaders said,

The BJ faculty is in contact with regional as well as national HIV-TB researches where a good amount of give and take of the information and the exploration of common areas where research can be done to get the Indian perspective regarding the management of various patients related to HIV-TB management.

#### Theme 4: BJGMC’s ability to disseminate research results

The baseline interviews confirmed the baseline survey findings that BJGMC capacity to publish and disseminate research results was limited. One of the TB experts said,

I’m not very familiar with that [TB research publications by BJGMC faculty]. I haven’t come across any [publication by BJGMC faculty].

All of the 26 participants in the follow-up interviews confirmed the quantitative survey findings that HIV-TB research publications had increased at BJGMC. One of the TB experts said,

I have seen the breadth and the number of the presentations, publications [by BJGMC faculty].

#### Theme 5: Barriers to conducting HIV-TB research at BJGMC

Although not addressed in the surveys, the baseline interviews identified barriers to conducting HIV-TB research at BJGMC as a key theme. Some bureaucratic barriers were also documented. One TB expert said,

The faculty are too busy doing clinical work… and [they have] very little opportunity or encouragement or incentives for research.

The follow-up interviews suggested that some administrative barriers to conducting HIV-TB research at BJGMC remain. One of the BJGMC leaders said,

The weakness is again…it’s not personal …individual weakness. It is a system’s weakness….it is hard for them to get this dedicated time because they are not only seeing patients, but they also have some administrative responsibilities…

Remaining barriers reported by Fogarty Scholars included lack of dedicated space for research and the lack of a research culture at this institution. One Fogarty Scholar said,

…when we are doing our [research] work, it would have been very nice if everybody was very supportive. Because being a very busy clinician, it may not be possible to take out a lot of time. But whatever time I have been able to give, I feel that administrative support should be much more. And when we are writing, I feel that everybody should write together.

An important administrative barrier mentioned by 4 of the 19 Fogarty Scholars interviewed was the risk that the government could transfer them to another institution, interrupting their research work. One of the Fogarty Scholars said,

…When we were transferred as professors out of the institute as a part of the Government rules, we had difficulty in conducting the study over here and giving our inputs…That, I feel, was a gap in the project…

#### Theme 6: Fogarty Scholars’ perspective regarding the Fogarty training program

At the evaluation interview, the Fogarty Scholars’ perspectives regarding their own research capacity development were also documented. One of the scholars revealed that the training program has changed the vision and cultivated research culture, enabling the streamlining of the guidelines for patient benefit. One Fogarty Scholar said,

Because previously you know HIV/TB patient used to come, we used to examine and we used to give treatment. Now I am collecting this data, and I am directing this patient, and keeping keen watch on this patient to improve their vision. So it has changed my view after attending this Fogarty Program, my mind has changed to research purpose more than treating the patient, just treating a person. So I take special care that how I should improve this patient and give more services to these HIV/TB patients.

One of the scholars also mentioned that it was a very good program that had led them to learn so many things and felt this should be a continuous process.

It was a good program. At international level, we have learned many things about the biostatistics and how to do the research, how to plan a research, which topics, how to select the research topic. Every alternate day, Dr. Bollinger sir taught us regarding the selection of the research topic, how to proceed for the research, and then how to compile the data, how to approach for the international publication in the PubMed journals. That was very…… it was very good experience for the research persons like me, it was very good experience. And I think Fogarty Program if possible must be continued here forwards.

#### Theme 7: Suggestions to overcome barriers to HIV-TB research

The follow-up interviews also identified a number of suggestions to overcome remaining barriers to conducting HIV-TB research at BJGMC. Two out of four independent TB experts interviewed mentioned that the ongoing research training was needed. One of the experts said,

… I think the capacity building is a long-term process, and it takes at least a minimum of 10 years I think to have some impact.

The most common suggestion, mentioned by 22 (85%) of the 26 interviewees, was to increase dedicated space and/or protected time for research at BJGMC. One of the Fogarty Scholars said,

Because of work pressure what happens here, people don’t respect the value of your time. And then you are constantly burdened with this and that and that. If somebody says, “I am giving this time to research”, it should be respected.

## Discussion

Some observations resulting from this evaluation study can serve as key “lessons learned” for the design and implementation of future research training programs. Our analyses demonstrated that the Fogarty program enhanced both the individual and institutional research capacity in a large public, government medical institution in India. We found that the number of research publications and IRB-approved research projects by the Fogarty Scholars notably increased after this training program. The scholars who were exposed for a longer duration to the Fogarty training program (according to their time of recruitment) contributed to a greater number of publications. Our first group of scholars in particular included individuals who had already worked with research groups based at their campus. In addition, NIH research funding supplement for the research study protocol designed by the group one Fogarty Scholars under the Fogarty training program is one of the major success indicators. These metrics reflected the improvement in individual research capacity development.

Fogarty Scholars were also able to engage other faculty colleagues from outside the training program in journal clubs, research methodology workshops, and their own research projects, demonstrating increased institutional research capacity. Moreover, the Fogarty Scholars’ students’ ability to obtain ICMR funding for their research projects illustrates the improvement of individual as well as institutional research capacity.

Baseline quantitative surveys and qualitative in-depth interviews also confirmed that the institution had a perceived need to increase research capacity in TB laboratory support, TB clinical research, Good Clinical Laboratory Practice (GCLP), TB epidemiology, and the ethical conduct of research and data management. Significant improvement in all these areas was reported at the end of the Fogarty training program. In addition, the program scholars identified nosocomial TB transmission as the primary concern faced by their students and colleagues, and consequently, all Group 1 scholars focused their research efforts on this issue [15,16,17,18]. Encouragingly, the program facilitated identification of ground-up capacity strengthening needs that resulted in regular TB screening of medical students and publication of these findings in international peer-reviewed journals. On the other hand, the Group 2 scholars undertook research in alternative priority domains, such as childhood tuberculosis [19] and child contact screening with isoniazid preventive treatment [20].

The survey results also highlighted an increase in the number of research publications and the collaboration of the BJGMC faculties with other researchers. However, in-depth interviews of scholars conveyed the necessity to further improve their manuscript writing skills in order to have more publications. Additionally, qualitative in-depth interviews also reported the need for dedicated time, space, and support from the administration for the sustenance of the research culture at the institution. The need to continue this training program for maintaining and consolidating the research culture at the institution has been reported by institutional leaderships and Fogarty Scholars.

Recently, Fogarty has expanded its support from individual to institutional research-capacity strengthening. Our program evaluation metrics were aligned with those recommended by FIC [21]. One previous evaluation study focused only on qualitative assessment [22]; however, our study integrated the qualitative assessment in support with quantitative measures to arrive at a strong conclusion.

Our assessment is consistent with most of the earlier program evaluations, which focused on individual research training outcomes in terms of publications, conference presentations, successful grant applications, qualifications obtained, and dissemination of study results [11,23,24,25,26,27,28,29], and for institutional capacity strengthening, the number of workshops and training activities at institutes [30].

However, one of the assessment studies has mentioned that publications in peer-reviewed journals are a long process for the low research skill base in some areas of health care practice [21], hence adequate time needs to be provided to evaluate the publication outcome. Our program showed an increase in publications from 18 at the start to 49 additional new publications by Fogarty Scholars at the end of the training program. These scientific publications underline the success of the training program and also showcase the strength and diversity of the research conducted by the Fogarty Scholars.

Compared to the previously reported evaluation by Zachariah R, et al. [29], our study not only documented the initiation of scientific research projects but also focused on the publication outcome of the scientific research in PubMed.

Developing research capacity in a specific disease is a well-established mechanism to stimulate the next generation of global health leaders [31]. One of the earlier program assessment studies has documented the reasons for the failure of research publications. It drew attention to factors such as lack of dedicated time and opportunity, wrong choice of research question, weak results due to poor study design, inadequate writing and language skills, peer review rejection fatigue, no ethics clearance, rapid staff turnover, disapproval from supervisors, lack of funding and infrastructure, and lack of leadership support [29]. In agreement with this, our qualitative interviews brought out more or less similar perceptions regarding the conduct of HIV-TB research and sustainability of research culture at the institution.

Measuring institutional research capacity strengthening is more challenging [27]. The knowledge gained by the beneficiaries needs to be shared and disseminated. This could be one of the important indicators to assess research training programs [29]. Our study demonstrated that Fogarty Scholars involved other colleagues and their mentees as co-authors in their publications, which in turn implied the dissemination and sharing of their research proficiency. Five students mentored by Fogarty Scholars received national funding for their research activity, and 14 of the other BJGMC faculties were listed as co-authors in the publications authored by Fogarty Scholars. Fogarty Scholars have initiated a yearly research methodology workshop activity for all under and postgraduate students. This is currently an ongoing process, which further emphasizes the need to sustain institutional research capacity strengthening.

This study also demonstrated that long-term research collaborations (i.e., with JHU) are not sufficient to ensure institutional research capacity is fully developed, and that government support is imperative to cultivate research culture at the institution. One study has suggested that making a small investment to retain talented, highly motivated scholars will be helpful for ensuring research capacity development [32]. We strongly believe that a similar strategy might be extrapolated to the Fogarty training program.

## Strength and Limitations

The study has a number of strengths. Most program evaluations have focused on individual metrics; this program focused on both individual as well as institutional research capacity. The mixed-methods approach allowed for enriched interpretation of the program evaluation findings. Support of qualitative data to quantitative survey findings helped reach a strong conclusion. One more strength of the evaluation is the inclusion of beneficiaries, that is, Fogarty Scholars’ opinions.

This study also has several limitations. Survey response rates at both time points (2014 and 2018) was less than 50%, which may result in bias for the quantitative results [33]. This evaluation accentuated the short-term impact, if we measure the impact at the end of the program, that is, at year five, we may not be able to estimate its sustainability. However, this may reveal a different impact five years after this evaluation (i.e., five years after the completion of the program). Our anonymous survey method was another limitation, as it did not allow for baseline versus year-five comparison from the same respondents. Inclusion of BJGMC leadership and scholars in follow-up surveys may have an impact on the follow-up survey results. While the input of BJGMC institutional leaders was certainly reflected in the design of the Fogarty training program, unfortunately this input was not collected using the standardized qualitative methods used for the baseline key informant interviews. Adding Fogarty Scholars as survey respondents at follow-up did not permit direct comparison of qualitative interview results at baseline and follow-up. Also, this addition of Fogarty Scholars at baseline and the anonymous nature of the data collection did not enable us to limit our statistical analysis to a comparison of the response rates to those that participated in both the baseline and follow-up surveys. This study also did not assess other metrics that could have indicated institutional growth, such as grant management offices and resources, improvements in ethics approval processes, and so on.

## Conclusion

The Fogarty training program had a significant impact on individual research-capacity development compared to indicators for institutional research-capacity strengthening. For both institutional and individual research-capacity development, sustained administrative support is important. Continuous research training and writing workshops to improve the scientific writing skills of faculty and students are required for viable research capacity strengthening at the institution. Sustainability of our program’s impact will likely depend on BJGMC’s institutional support, in terms of protected time for research, space, and meaningful incentive to develop research independence.

## Data Accessibility Statement

The datasets used and/or analyzed during the current study are available from the corresponding author on reasonable request.

## Additional Files

The additional files for this article can be found as follows:

10.5334/aogh.2932.s1 Annexure I. Survey questionnaire.

10.5334/aogh.2932.s2 Annexure II A. Interview guide_Expert.

10.5334/aogh.2932.s3 Annexure II B. Interview guide_Leaders.

10.5334/aogh.2932.s4 Annexure II C. Interview guide_Scholars.

## Abbreviations

JHU: Johns Hopkins University; BJGMC: Byramjee Jeejeebhoy Government Medical College; TB: Tuberculosis; FIC: Fogarty International Center; NIH: National Institutes of Health; GCP: Good Clinical Practice; IDIs: in-depth interviews; ICMR: Indian Council of Medical Research; USA: United State of America; PLHIV: People Living with HIV; LMIC: Low- and middle-income countries.
