# Supplementary material for: Impact of the Fogarty Training Program on Trainee and Institutional Research Capacity Building at a Government Medical College in India
Source: Ann Glob Health. 2020 Jul 28;86(1):86. doi: 10.5334/aogh.2932 (PMC7394206; doi:10.5334/aogh.2932)
Supplement: Annexure I. — Survey questionnaire. [file agh-86-1-2932-s1.pdf]

BJGMC-JHU FOGARTY
HIV-TB TRAINING PROGRAM

Introduction

In August 2013, a five-year Fogarty HIV TB Training grant was awarded to Johns Hopkins University (JHU) in partnership with the Byramjee Jeejeebhoy Government Medical College (BJGMC). The purpose of the program was to increase institutional capacity for conducting HIV-TB-focused research by establishing a cadre of highly trained faculty who are capable of building BJGMC's expertise in these disciplines.

After nearly five years of training efforts, we ask you to aid us as we evaluate the impact of our program. We invite you to be part of a survey that was administered at baseline, and is being repeated to assess for changes over time. The survey is 15 questions long and should take you less than 15 minutes to complete. Please reflect on the current individual and institutional strengths and weaknesses for doing HIV-TB research at BJGMC. Your responses will be anonymized, and responses analyzed in aggregate. Your completion of this survey will serve as consent to take part in a research study to evaluate BJGMC's capacity. Thank you in advance for your time.

Over the past year, how often have you interacted with HIV-TB experts based at BJGMC medical college?

- ○ Always, more than once a month
- ○ Often, more than once every few months
- ○ Sometimes, a handful of times a year
- ○ Rarely, only a few times per year
- ○ Never

Please take a moment to rank the strengths of HIV-TB researchers at BJGMC:

	Very strong, no room for improvement	Strong, with some room for improvement	Mediocre	Weak, with much room for improvement	Very weak, only way to go is up
Basic research knowledge	○	○	○	○	○
TB laboratory skills	○	○	○	○	○

	Very strong, no room for improvement	Strong, with some room for improvement	Mediocre	Weak, with much room for improvement	Very weak, only way to go is up
TB clinical knowledge	○	○	○	○	○
Knowledge of the epidemiology of TB	○	○	○	○	○
Scientific output (journal articles)	○	○	○	○	○
Linkage with government	○	○	○	○	○

What areas require the most training?

	Strongest need for training	Strong need for training with some existing expertise	Moderate need for training with good expertise established	Weak need for training with very good expertise established	Weakest need for training
Basic research knowledge	○	○	○	○	○
TB laboratory skills	○	○	○	○	○
TB clinical knowledge	○	○	○	○	○
Knowledge of the epidemiology of TB	○	○	○	○	○
Scientific output (journal articles)	○	○	○	○	○

	Strongest need for training	Strong need for training with some existing expertise	Moderate need for training with good expertise established	Weak need for training with very good expertise established	Weakest need for training
Linkage with government	○	○	○	○	○
Other, specify: ________	○	○	○	○	○

Over the past year, how would you rank the quality of TB research input and output from BJGMC researchers?

	Excellent	Good	Average	Poor	Terrible	Not enough information to answer
Design of TB research studies	○	○	○	○	○	○
Implementation of TB research studies	○	○	○	○	○	○
Lectures that disseminate findings of research	○	○	○	○	○	○
Abstracts that disseminate findings of research	○	○	○	○	○	○
Articles that disseminate findings of research	○	○	○	○	○	○

	Excellent	Good	Average	Poor	Terrible	Not enough information to answer
Linkage to policy makers and other stakeholders	○	○	○	○	○	○

Please use the box below to comment on any additional skills and/or knowledge gaps that you see among individual TB researchers at BJGMC:

→

Powered by Qualtrics

The following questions are meant to asses the institutional capacity of BJGMC, not individual capacity. Please answer the questions below as you consider the overall capacity of BJGMC to do TB research.

How well positioned is BJGMC to do TB research with regard to the following:

	Very well positioned, no gaps	Well positioned with some institutional gaps	Somewhat well positioned with institutional gaps	Not very well positioned with significant institutional gaps	Badly positioned with major institutional gaps	Not enough experience to say
TB laboratory facilities	○	○	○	○	○	○
Good Clinical Laboratory Practice	○	○	○	○	○	○

	Very well positioned, no gaps	Well positioned with some institutional gaps	Somewhat well positioned with institutional gaps	Not very well positioned with significant institutional gaps	Badly positioned with major institutional gaps	Not enough experience to say
TB clinical research systems (specimen collection, CXRs, proper clinical care)	○	○	○	○	○	○
TB epidemiological investigations (surveillance systems, patient data)	○	○	○	○	○	○
Ethical conduct of research	○	○	○	○	○	○
Data management systems	○	○	○	○	○	○
Research dissemination systems	○	○	○	○	○	○

How likely is BJGMC's TB research group to:

	Not at all likely	Slightly likely	Moderately likely	Very likely	Completely likely

	Not at all likely	Slightly likely	Moderately likely	Very likely	Completely likely
Empower young faculty to build their careers	○	○	○	○	○
Carry out the technical requirements for research	○	○	○	○	○
Carry out the logistical requirements for research	○	○	○	○	○
Attract additional funding for TB research	○	○	○	○	○
Dedicate and/or increase space for TB research	○	○	○	○	○
Retain and promote young faculty focused on TB research	○	○	○	○	○
Adapt to TB laboratory advances	○	○	○	○	○
Adapt to TB clinical advances	○	○	○	○	○
Encourage innovation	○	○	○	○	○

How would you rate BJGMC's TB research linkages to:

	Excellent	Good	Fair	Poor	Terrible	Not enough information to answer
Other BJGMC researchers	O	O	O	O	O	O
BJGMC medical students	O	O	O	O	O	O
Government bodies, including the RNTCP	O	O	O	O	O	O
Other Indian TB researchers	O	O	O	O	O	O
Other Foreign TB researchers	O	O	O	O	O	O
Other, please specify:	O	O	O	O	O	O

Powered by Qualtrics

This is the final section of the survey, and is meant to provide open feedback on how you view the successes and failures of the program, and insights for future training programs.

In your opinion, what were the successes of the BJGMC-JHU Fogarty program?

Do you think the BJGMC-JHU Fogarty program was effective as a way to build institutional capacity for HIV-TB research?

- Extremely effective
- Very effective
- Moderately effective
- Slightly effective
- Not effective at all

In your opinion, what were the failures of the BJGMC-JHU Fogarty program?

What advice would you give for future training efforts of government medical faculty in India for building HIV-TB research capacity?

Did you participate in the baseline survey in 2014?

- Yes
- No
- Can't remember

In order to help stratify our results by exposure to program activities through RePORT-India, please indicate whether you are aware of program activities only because of your participation in the RePORT-India network.

- Yes
- No

In the baseline survey, we had asked for details on your position. Kindly enter this information so that we can provide information on the baseline and final survey populations. Thank you again for your help in evaluating our program.

What is your position:

- ○ Senior faculty at BJGMC
- ○ Junior faculty at BJGMC
- ○ Stakeholder at BJGMC (non-faculty)
- ○ Researcher/Scientist
- ○ Government Employee
- ○ Please leave your email if you are willing to have your baseline responses evaluated in comparison to our final evaluation.

→

Powered by Qualtrics
