# Supplementary material for: Impact of the Fogarty Training Program on Trainee and Institutional Research Capacity Building at a Government Medical College in India
Source: Ann Glob Health. 2020 Jul 28;86(1):86. doi: 10.5334/aogh.2932 (PMC7394206; doi:10.5334/aogh.2932)
Supplement: Annexure II A. — Interview guide_Expert. [file agh-86-1-2932-s2.pdf]

Key Informant Interview Guide (For Experts)
BJGMC JHU Fogarty HIV TB Training Program, Pune, India

1.0 Introductions

Thank you for meeting with me today. The reason you've been contacted to participate in this study is because of your standing as a research expert who will help the investigators of the BJGMC-JHU Fogarty program understand your views on BJGMC's capacity to do TB research.

We'd like to know more about your impression of the parameters required for BJ to be considered as center for HIV TB research, as well as some of the barriers to the institution's growth in this regard.

Before we begin, I want to make sure that you understand what this study involves.

Go over Informed Consent form:

- *This form explains how we are doing our research. The purpose of this form is to help you decide whether you want to be interviewed or not.*
- *The interview will last about an hour and it will be audio recorded. We will talk about BJGMC's capacity to do HIV TB research. You don't need to answer any question and you can stop the interview at any time.*
- *We will be using the information from the interview to help us to assess the program's performance over time and success in reaching program goals*
- *Information from this interview will remain confidential.*

2.0 Warm-up

Please tell me about your background in HIV TB research (please go over the questions listed in the table).

Current position	Years in HIV TB research	Institution	Any collaboration with BJ faculty	Any collaboration with Johns Hopkins faculty	Any collaboration with national/international investigators (ICMR, ISER, etc? Others)

3.0 Ability of BJGMC faculty to do HIV TB research

3.1: What is your impression of the BJGMC faculty's ability to do HIV TB research?

3.2: Please update me whom you have seen more often in HIV TB research from BJGMC.

3.3: What are some of the institution's strengths with regard to doing HIV TB research?

3.4: What are some of the institutions' weaknesses with regard to doing HIV TB research?

3.5: Please let us know your suggestions / recommendations to overcome this weaknesses?

3.6: Do you think, in general, that other HIV TB researchers in India see BJGMC as an institution that has the facilities and expertise to conduct quality research? If yes/no, reasons?

3.7: Do you think that BJGMC's ability to do research has any dependence on their partnership with Johns Hopkins/other institutions?

4.0 Ability of BJGMC to disseminate results of HIV TB research

4.1: Have you had any experience with HIV TB research publications from BJGMC faculty as lead/senior author for work done at the institution/associated hospital? How was your experience?

4.2: Can you recall any conference presentations on HIV TB from BJGMC faculty? Which was that (we can expect broad theme instead of specific topic eg HIV TB)? Name of the conference, How was the presentation? What are your views on the same?

4.3: From BJGMC collaborators, what HIV TB research publications you have seen? Which are those, what is your view/opinion on these publication?

4.4: From BJGMC collaborators for work done at the institution/associated hospital, what are the HIV TB conference presentations you have seen?

4.5: In general do you feel that the HIV TB scientific community sees publications/research news from BJGMC? In what format?

4.6: What are some of the barriers to study dissemination that the institution/collaborators might face? Eg. Authorship guidelines, etc.

4.7: Please provide your suggestions to overcome these barriers.

5.0 Ability of BJGMC faculty to attain funding for HIV TB research

5.1: What are your views or opinion for BJGMC faculty's success at attaining funding for HIV TB research?

5.2: Similarly, what is your opinion about BJGMC faculties' capabilities of independently attaining future funding for HIV TB research as lead investigators?

5.3: Could you please provide your suggestions to overcome these barriers?

6.0 Ability of BJGMC faculty to collaborate with other HIV TB research stakeholders

6.1: How do you view the ability of BJGMC faculty to collaborate with regional/national HIV TB investigators?

6.2: What are your views on the government structure for building institutional collaborations?

6.3: What are some of the barriers to BJGMC building collaborative relationships with other groups/institutions?

6.4: Could you please provide your suggestions to overcome these barriers?

7.0 Changes over the course of the Fogarty program

7.1: Were you aware of any HIV TB research at BJGMC started in 2013? (if no, skip)

7.2: Were you aware that the BJGMC Fogarty program had received funding from the NIH for research capacity building (if no, skip)

7.3: What do you think the Fogarty program has done for BJGMC research capacity over five years?

7.4: Due to Fogarty Program, What changes, positive or negative, have you seen in BJGMC HIV TB research output over the past five years?

7.5: What are the changes, you have observed in BJGMC faculty or Government of India employees towards research?

7.6: Have you seen BJGMC faculty, or Government of India employees, produce more or less research since you became aware of the program?

7.7: Given your historical exposure to our program, what do you see as some of the greatest needs for increasing the institution's capacity to do research that are still outstanding?

7.8: What recommendations would you make for future research capacity building efforts at BJGMC?

7.9: What recommendations would you make for future research capacity building efforts in India?

7.10: What is your opinion on beneficiaries? What do you think, whom should be the beneficiaries of this program?

7.11: What are your views for cultivation of research among UG and PG students and creating awareness among them about research ethical guidelines?

8.0 Conclusion

8.1: What are your suggestions or recommendations for sustainability of Fogarty program at BJGMC?

8.2: Is there any other information that you think we should know about the capacity of BJGMC to conduct HIV TB research, or increasing the institution's ability?

8.3: Do you have any questions about this interview, or what we talked about?

Thank you for sharing your time and insights today.
