# Supplementary material for: Impact of the Fogarty Training Program on Trainee and Institutional Research Capacity Building at a Government Medical College in India
Source: Ann Glob Health. 2020 Jul 28;86(1):86. doi: 10.5334/aogh.2932 (PMC7394206; doi:10.5334/aogh.2932)
Supplement: Annexure II C. — Interview guide_Scholars. [file agh-86-1-2932-s4.pdf]

Key Informant Interview Guide (For Scholars)
BJGMC JHU Fogarty HIV TB Training Program, Pune, India

1.0 Introductions

Thank you for meeting with me today. The reason you've been contacted to participate in this study is because of your standing as a research expert who will help the investigators of the BJGMC-JHU Fogarty program understand your views on BJGMC's capacity to do TB research.

We'd like to know more about your impression of the parameters required for BJ to be considered as center for HIV TB research, as well as some of the barriers to the institution's growth in this regard.

Before we begin, I want to make sure that you understand what this study involves.

Go over Informed Consent form:

- *This form explains how we are doing our research. The purpose of this form is to help you decide whether you want to be interviewed or not.*
- *The interview will last about an hour and it will be audio recorded. We will talk about BJGMC's capacity to do HIV TB research. You don't need to answer any question and you can stop the interview at any time.*
- *We will be using the information from the interview to help us to assess the program's performance over time and success in reaching program goals*
- *Information from this interview will remain confidential.*

2.0 Warm-up

Please tell me about your background in HIV TB research (please go over the questions listed in the table).

Current position	Years in HIV TB research	Institution	Any collaboration with Johns Hopkins faculty	Any collaboration with national/international investigators (ICMR, ISER, etc? Others)

3.0 Views regarding Fogarty program

3.1: Please let us know your views regarding Fogarty Program.

3.2: Do you think this program helps you to develop your HIV TB capacity? How this program helps you to do so.

3.3: Do you think this program helps the BJGMC institution to become a leader in HIV TB research in India? Yes/No, how and why

3.4: Kindly narrate your barriers/challenges to complete the Fogarty scholarship program, how did you overcome those?

3.5: Kindly narrate your achievements due to Fogarty program.

3.6: How this program does helps you, or your institution to do HIV TB research in India

3.7: What is your view regarding sustainability of Fogarty Program at BJGMC?

3.8: What are your suggestions to sustain this program at BJGMC and who will support to sustain the program?
